# Diagnostic value of alpha-fetoprotein combined with neutrophil-to-lymphocyte ratio for hepatocellular carcinoma

**DOI:** 10.1186/s12876-018-0908-6

**Published:** 2018-12-13

**Authors:** Jian Hu, Nianyue Wang, Yongfeng Yang, Li Ma, Ruilin Han, Wei Zhang, Cunling Yan, Yijie Zheng, Xiaoqin Wang

**Affiliations:** 1 Department of Clinical Laboratory, The First Affiliated Hospital of Xi’an Jiaotong University, 277 West Yanta Road, Xi’an, 710061 People’s Republic of China; 2 Department of Clinical Laboratory and Liver Diseases, The Second Hospital of Nanjing, Affiliated to Medical School of Southeast University, Nanjing, 210000 China; 3 Department of Clinical Laboratory, Peking University First Hospital, Beijing, 100000 China; 4 Department of Mathematics & Statistics, University of Arkansas at Little Rock, Little Rock, AR 72204 USA; 5 Medical Scientific Affairs, Abbott Diagnostics Division, Abbott Laboratories, Shanghai, 200032 China

**Keywords:** Alpha-fetoprotein, Neutrophil-granulocyte ratio, Hepatocellular carcinoma

## Abstract

**Background:**

To investigate the diagnostic performance of alpha-fetoprotein (AFP) and neutrophil-to-lymphocyte ratio (NLR) as well as their combinations with other markers.

**Methods:**

Serum aspartate aminotransferase (AST), alanine aminotransferase (ALT), AFP and levels as well as the numbers of neutrophils and lymphocytes of all enrolled patients were collected. The NLR was calculated by dividing the number of neutrophils by the number of lymphocytes. Receiver operating characteristic (ROC) curve analysis was conducted to determine the ability of each marker and combination of markers to distinguish HCC and liver disease patients.

**Results:**

In total, 545 patients were included in this study. The area under the ROC curve (AUC) values for AFP, ALT, AST, and NLR were 0.775 (0.738–0.810), 0.504 (0.461–0.547), 0.660 (0.618–0.699), and 0.738 (0.699–0.774) with optimal cut-off values of 24.6 ng/mL, 111 IU/mL, 27 IU/mL, and 2.979, respectively. Of the four biomarkers, AFP and NLR showed comparable specificity (0.881 and 0.858) and sensitivity (0.561 and 0.539). The combination of AFP and NLR showed the highest AUC (0.769) with a significantly higher sensitivity (0.767) and a lower specificity (0.773) compared to AFP or NLR alone, and it had the highest sum of sensitivity and specificity (1.54) among all combinations. In patients with AFP < 20 ng/mL, the NLR showed the highest AUC and combination with other markers did not improve the diagnostic accuracy.

**Conclusions:**

Our data indicate that the combination of AFP and NLR offers better diagnostic performance than either marker alone for differentiating HCC from liver disease, which may benefit clinical screening.

## Background

Liver cancer is the sixth most common cancer and the third leading cause of cancer-related death worldwide [1, 2]*.* Hepatocellular carcinoma (HCC), which accounts for 70–85% of liver cancer cases, is always diagnosed in an advanced stage and is associated with a poor prognosis, with a 5-year overall survival rate of less than 15% [3, 4]. At present, treatments such as surgery and liver transplantation for early-stage HCC result in better outcomes with a 5-year overall survival rate of more than 70% [5–7]. Therefore, diagnosis of HCC during an early stage is pivotal for improving the clinical outcomes of patients.

Alpha-fetoprotein (AFP) is the most widely used serum marker for screening and initial diagnosis of HCC in clinical practice. However, the sensitivity of AFP is only about 60% at a cut-off value of 20 ng/mL, and the specificity is low [8–10]. Moreover, AFP levels remain normal in 15–30% of patients with advanced stage disease and increase in some patients with chronic hepatitis, liver cirrhosis, and other liver diseases [4, 11], leading to high negative and false-positive rates. Therefore, novel markers that complement the limitations of AFP are needed to for screening and more accurate diagnosis of HCC.

Crosstalk between cancer cells and their inflammatory microenvironment plays critical roles in the initiation and progression of cancer, including the promotion of angiogenesis, proliferation, and metastasis [12–14]. Inflammatory infiltrates in the tumor microenvironment largely influence the biological behavior of HCC [15–18]. The neutrophil-to-lymphocyte ratio (NLR) is one parameter reflecting the presence of a systemic inflammatory response and can be readily determined at low cost through routine blood examination. The baseline NLR has been reported to be a valuable predictor in many cancers, including colorectal cancer [19], renal cancer [20], diffuse large B-cell lymphoma [21, 22], and HCC [23]. The NLR also has been reported to be diagnostic marker for peptic ulcer perforation [24], acute mesenteric ischemia [25], and lung cancer [26, 27].

Thus, we questioned whether the NLR can be used as a supplementary diagnostic marker with AFP. This study aimed to evaluate the diagnostic value of AFP in combination with the NLR for HCC. To better investigate the relative diagnostic value of serum biomarkers, two common serum biomarkers for liver function, aspartate aminotransferase (AST) and alanine aminotransferase (ALT), also were analyzed in the present study.

## Methods

### Patients

Patients diagnosed with HCC and liver disease were enrolled at the three centers (Peking University 1st Hospital, Xi’an Jiaotong University 1st Hospital and The Second Hospital of Nanjing, Affiliated to Medical School of Southeast University) between July 2013 and July 2016. HCC was diagnosed according to the Asian Pacific Association for the Study of the Liver (APASL) consensus recommendations on HCC [28]. Only newly diagnosed and treatment-naïve patients with HCC were enrolled in the present study. Liver disease samples were mainly from patients infected with hepatitis B virus (HBV) or hepatitis C virus (HCV) and include samples from patients with hepatitis and cirrhosis, which were diagnosed according to APASL guideline.

This study was conducted according to the Declaration of Helsinki and approved by the Ethics of Committee.

### Data collection

Serum AST and ALT concentrations and the numbers of neutrophils and lymphocytes were recorded from routine clinical testing. Serum AFP was measured using the Abbott ARCHITECT hepatitis B surface antigen chemiluminescent microparticle immunoassay (Abbott Diagnostics, Abbott Park, IL, USA). The NLR was calculated by dividing the number of neutrophils by the number of lymphocytes.

### Statistical analysis

All statistical analyses were performed using SPSS (version 21; IBM, Armonk, NY, USA). Data are presented as mean ± standard derivation for normally distributed continuous data, as median (interquartile range, Q25–Q75) for abnormally distributed continuous data, or as actual values for categorical data. Comparisons between two groups were performed using *t* test, Wilcoxon test, or chi-square test. Receiver operation characteristic (ROC) curves were used to compare the diagnostic performance of each biomarker. The area under the ROC curve (AUC) for each biomarker for distinguishing HCC and liver disease patients as well as the optimal cut-off value, sensitivity, specificity, positive predictive value (PPV), and negative predictive value (NPV) were calculated using MedCalc. Combinations of markers were analyzed, and the related parameters were calculated with the online statistical software OpenEpi (http://www.openepi.com/Menu/OE_Menu.htm). A *P* value < 0.05 was considered to indicate a statistically significant difference.

## Results

### Clinical characteristics of the participants

In total, 545 patients, 369 with HCC and 176 with liver disease (21 cases of cirrhosis, 130 cases of hepatitis, and 25 cases of other diseases including autoimmune liver disease and alcoholic liver disease) were included in this study. The clinical characteristics of all participants are shown in Table 1. While ALT levels did not differ significantly, HCC patients were older, more likely to be male, had fewer neutrophils and lymphocytes, had a higher NLR, and had higher AST and AFP levels than liver disease patients (all *P* < 0.001).

**Table 1:** Clinical characteristics of the patients

	Liver disease (*n* = 176)	HCC (*n* = 369)	*P* value
Age (y)	46.34 ± 11.71	56.91 ± 10.04	< 0.001
Gender (M/F)	124/52	318/51	< 0.001
Neutrophils (× 10^9^/L)	5.705 (2.97–58.20)	0.674 (0.57–0.78)	< 0.001
Lymphocytes (× 10^9^/L)	3.01 (1.65–30.85)	0.209 (0.12–0.295)	< 0.001
NLR	1.851 (1.43–2.53)	3.23 (1.91–6.62)	< 0.001
ALT (IU/mL)	38.5 (26.00–66.50)	38.9 (23.75–73.93)	0.739
AST (IU/mL)	29 (21.00–52.00)	44 (29.78–85.65)	< 0.001
AFP (ng/mL)	3.67 (2.43–10.36)	41.16 (5.59–1030.03)	< 0.001

HCC, hepatocellular carcinoma; M, male; F, female; NLR, neutrophil-granulocyte ratio; ALT, alanine aminotransferase; AST, aspartate aminotransferase; AFP, alpha-fetoprotein

Data are presented as mean ± standard derivation for age, actual values for gender, and median (interquartile range, Q25-Q75) for other parameters

Comparisons between two groups were performed using *t* test for age, chi-square test for gender, or Wilcoxon test for other parameters

### Diagnostic accuracy of serum biomarkers for detecting HCC

**Fig. 1:**
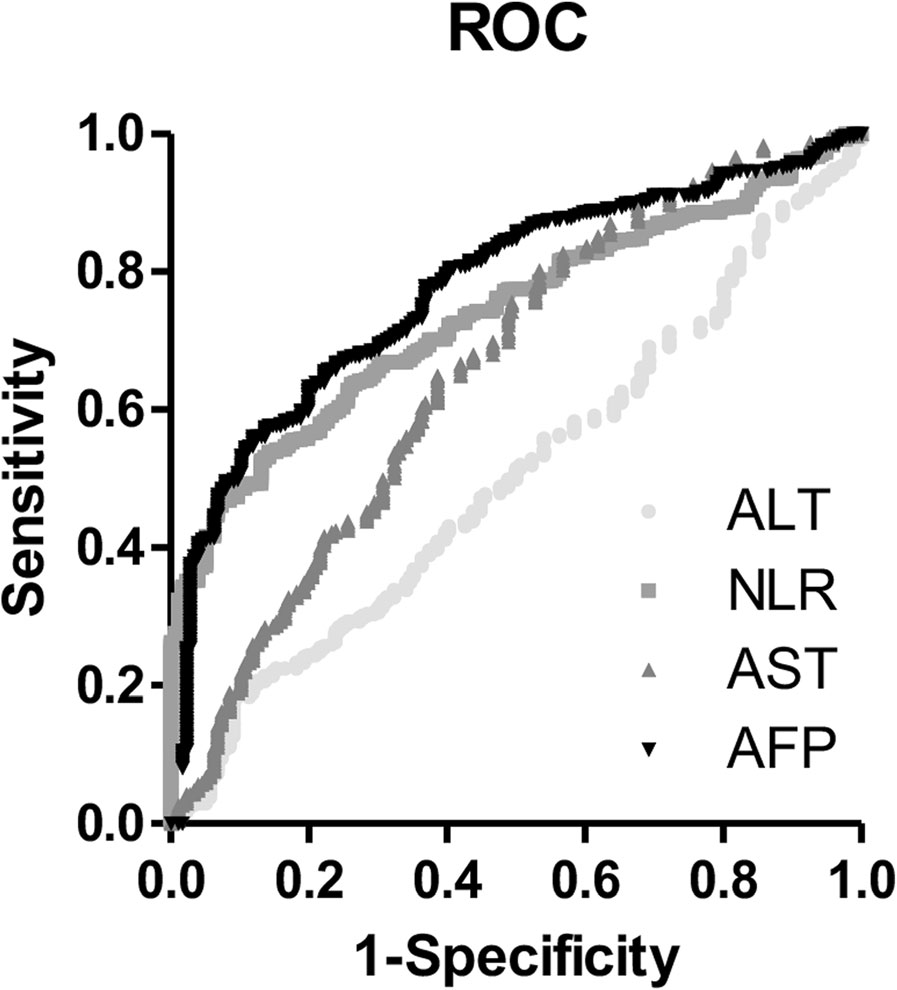
ROC curves for AFP, NLR, AST and ALT for the diagnosis of HCC with liver disease control

The ROC curves for serum biomarkers (AFP, ALT, AST, and NLR) for diagnosing HCC are shown in Fig. 1. The AUC values for AFP, ALT, AST, and NLR were 0.775 (0.738–0.810), 0.504 (0.461–0.547), 0.660 (0.618–0.699), and 0.738 (0.699–0.774) with optimal cut-off values of 24.6 ng/mL, 111 IU/mL, 27 IU/mL, and 2.979, respectively. When applying the common cutoff value of 20 ng/mL for AFP, the AUC was 0.664 (0.6224–0.703) (Table 2). Of the four biomarkers, ALT showed the highest specificity (0.909) with the lowest sensitivity (0.184), and AFP and NLR individually showed both higher specificity (0.881 and 0.858) and higher sensitivity (0.561 and 0.539) compared to AST (Table 2).

**Table 2:** Diagnostic performances of four serum biomarkers for differentiating HCC from liver disease

Marker	Cutoff value	AUC	Sensitivity (Sn)	Specificity (Sp)	Sn + Sp	PPV	NPV
ALT	111	0.504 (0.461–0.547)	0.184 (0.146–0.228)	0.909 (0.857–0.947)	1.093	0.810 (0.709–0.887)	0.347 (0.304–0.392)
AST	27	0.660 (0.618–0.699)	0.802 (0.758–0.842)	0.466 (0.391–0.542)	1.268	0.759 (0.713–0.801)	0.529 (0.447–0.610)
AFP	24.64	0.775 (0.738–0.810)	0.561 (0.509–0.612)	0.881 (0.823–0.925)	1.442	0.908 (0.863–0.942)	0.489 (0.433–0.545)
	20	0.664 (0.624–0.703)	0.577 (0.525–0.628)	0.852 (0.791–0.901)	1.429	0.891 (0.845–0.928)	0.490 (0.433–0.548)
	40	0.633 (0.592–0.672)	0.501 (0.449–0.554)	0.903 (0.850–0.943)	1.404	0.916 (0.869–0.950)	0.464 (0.410–0.518)
	100	0.514 (0.467–0.560)	0.442 (0.390–0.494)	0.938 (0.891–0.968)	1.38	0.937 (0.890–0.968)	0.445 (0.393–0.497)
	200	0.580 (0.538–0.621)	0.396 (0.345–0.448)	0.960 (0.920–0.984)	1.356	0.954 (0.908–0.981)	0.431 (0.382–0.482)
	188.4	0.585 (0.544–0.626)	0.409 (0.359–0.461)	0.955 (0.912–0.980)	1.364	0.950 (0.903–0.978)	0.435 (0.385–0.486)
NLR	2.979	0.738 (0.699–0.774)	0.539 (0.487–0.591)	0.858 (0.797–0.906)	1.397	0.888 (0.840–0.926)	0.470 (0.415–0.527)

HCC, hepatocellular carcinoma; ALT, alanine aminotransferase; AST, aspartate aminotransferase; AFP, alpha-fetoprotein; NLR, neutrophil-granulocyte ratio; AUC, area under the receiver operation characteristics curve

Because both AFP and NLR showed low sensitivity values, the diagnostic value of biomarker combinations was evaluated. The AUC, sensitivity, specificity, PPV, and NPV as well as optimal cut-off values for each marker and different combinations of biomarkers are summarized in Table 3. As diagnostic biomarkers for HCC, among all combinations of two biomarkers, the combination of AFP and NLR showed the highest AUC (0.769) with a significantly higher sensitivity (0.767) and a lower specificity (0.773) compared to AFP or NLR alone. In addition, this combination had the highest sum of sensitivity and specificity (1.54) among all the two-marker combinations. However, the combination of NLR and AST was the most sensitive (0.892) with a specificity of 0.409. Among all combinations with three biomarkers, the combination of AFP, NLR, and AST was the most sensitive (0.927) with a specificity of 0.409 and showed the same accuracy as the combination of all four biomarkers (Table 2). The combination of AFP, NLR, and ALT showed the highest AUC (0.773) with the highest sum of sensitivity and specificity (1.524) among all the three-marker combinations.

**Table 3:** Diagnostic performances of combinations of four serum biomarkers for differentiating HCC from liver disease

Markers	AUC	Sensitivity (Sn)	Specificity (Sp)	Sn + Sp	PPV	NPV
AFP + NLR	0.769 (0.732–0.802)	0.767 (0.721–0.807)	0.773 (0.705–0.828)	1.54	0.876 (0.836–0.908)	0.613 (0.547–0.674)
AFP + ALT	0.697 (0.657–0.734)	0.639 (0.578–0.677)	0.841 (0.780–0.888)	1.48	0.892 (0.849–0.924)	0.519 (0.461–0.577)
AFP + AST	0.749 (0.711–0.783)	0.884 (0.847–0.912)	0.466 (0.394–0.540)	1.35	0.776 (0.734–0.813)	0.656 (0.569–0.734)
ALT+AST	0.694 (0.654–0.731)	0.802 (0.759–0.840)	0.470 (0.394–0.540)	1.272	0.759 (0.714–0.799)	0.529 (0.451–0.606)
NLR + ALT	0.653 (0.612–0.692)	0.596 (0.545–0.645)	0.773 (0.705–0.828)	1.369	0.846 (0.797–0.885)	0.477 (0.420–0.535)
NLR + AST	0.736 (0.697–0.771)	0.892 (0.856–0.919)	0.409 (0.339–0.483)	1.301	0.760 (0.717–0.798)	0.643 (0.551–0.726)
AFP + AST + ALT	0.749 (0.711–0.783)	0.884 (0.847–0.912)	0.466 (0.394–0.540)	1.35	0.776 (0.734–0.813)	0.656 (0.569–0.734)
NLR + AST + ALT	0.736 (0.697–0.771)	0.892 (0.856–0.919)	0.409 (0.339–0.483)	1.301	0.760 (0.717–0.798)	0.643 (0.551–0.726)
AFP + NLR + ALT	0.773 (0.735–0.806)	0.791 (0.747–0.830)	0.733 (0.663–0.793)	1.524	0.861 (0.821–0.894)	0.626 (0.558–0.689)
AFP + NLR + AST	0.760 (0.722–0.796)	0.927 (0.896–0.949)	0.409 (0.339–0.483)	1.336	0.767 (0.725–0.804)	0.727 (0.632–0.805)
AFP + AST + ALT+NLR	0.760 (0.722–0.796)	0.927 (0.896–0.949)	0.409 (0.339–0.483)	1.336	0.767 (0.725–0.804)	0.727 (0.632–0.805)

HCC, hepatocellular carcinoma; ALT, alanine aminotransferase; AST, aspartate aminotransferase; AFP, alpha-fetoprotein; NLR, neutrophil-granulocyte ratio; AUC, area under the receiver operating characteristic curve

For combinations of markers, similar results were obtained when applying the common cutoff value of 20 ng/mL AFP or the optimal cutoff value of 24.6 ng/mL AFP (Table 4). When used in combination, AFP and NLR showed the highest sum of sensitivity and specificity (1.511).

**Table 4:** Diagnostic performances of combinations of four serum biomarkers for differentiating HCC from liver disease using AFP = 20 ng/mL as cutoff value

Markers	AUC	Sensitivity (Sn)	Specificity (Sp)	Sn + Sp	PPV	NPV
AFP + NLR	0.762 (0.724–0.795)	0.772 (0.727–0.812)	0.739 (0.669–0.798)	1.511	0.861 (0.820–0.894)	0.608 (0.541–0.671)
AFP + ALT	0.697 (0.657–0.734)	0.694 (0.654–0.731)	0.807 (0.742–0.858)	1.501	0.874 (0.829–0.909)	0.516 (0.4458–0.575)
AFP + AST	0.749 (0.711–0.783)	0.889 (0.853–0.917)	0.470 (0.394–0.540)	1.359	0.777 (0.735–0.813)	0.667 (0.579–0.744)
ALT+AST	0.694 (0.654–0.731)	0.802 (0.759–0.840)	0.470 (0.394–0.540)	1.272	0.759 (0.714–0.799)	0.529 (0.451–0.606)
NLR + ALT	0.653 (0.612–0.692)	0.596 (0.545–0.645)	0.773 (0.705–0.828)	1.369	0.846 (0.797–0.885)	0.477 (0.420–0.535)
NLR + AST	0.736 (0.697–0.771)	0.892 (0.856–0.919)	0.409 (0.339–0.483)	1.301	0.760 (0.717–0.798)	0.643 (0.551–0.726)
AFP + AST + ALT	0.749 (0.711–0.783)	0.889 (0.853–0.917)	0.470 (0.394–0.540)	1.359	0.777 (0.735–0.813)	0.667 (0.579–0.744)
NLR + AST + ALT	0.736 (0.697–0.771)	0.892 (0.856–0.919)	0.409 (0.339–0.483)	1.301	0.760 (0.717–0.798)	0.643 (0.551–0.726)
AFP + NLR + ALT	0.765 (0.728–0.799)	0.799 (0.759–0.835)	0.699 (0.627–0.762)	1.498	0.847 (0.806–0.881)	0.621 (0.552–0.686)
AFP + NLR + AST	0.762 (0.724–0.795)	0.930 (0.899–0.952)	0.409 (0.339–0.484)	1.339	0.767 (0.73–0.804)	0.736 (0.640–0.812)
AFP + AST + ALT+NLR	0.762 (0.724–0.795)	0.930 (0.8998–0.952	0.409 (0.339–0.484)	1.339	0.767 (0.73–0.804)	0.736 (0.640–0.812)

HCC, hepatocellular carcinoma; ALT, alanine aminotransferase; AST, aspartate aminotransferase; AFP, alpha-fetoprotein; NLR, neutrophil-granulocyte ratio; AUC, area under the receiver operating characteristic curve

### Diagnostic accuracy of AFP with different cut-off value as well as in combination with three other biomarkers

Next, the sensitivity, specificity, PPV, and NPV for AFP at different cutoff values were analyzed. As shown in Table 2, with the increase in cutoff value, AFP showed decreased sensitivity (from 0.577 at 20 ng/mL to 0.396 at 200 ng/mL) and increased specificity (from 0.852 at 20 ng/mL to 0.955 at 200 ng/mL), with the highest AUC of 0.775 at 24.64 ng/mL. Then we used the cutoff value of AFP with 95% specificity, which was 188.40 ng/mL, in combination with other markers. If the sensitivity was as high as 90%, the cut-off values for AST, ALT, and NLR were 26.4–26.6, 20.8, and 1.7, respectively, and the cutoff values for AST and ALT were 29.6 IU/mL and 23.5 IU/mL.

### Diagnostic accuracy of NLR in patients with low AFP (< 20 ng/mL)

**Fig. 2:**
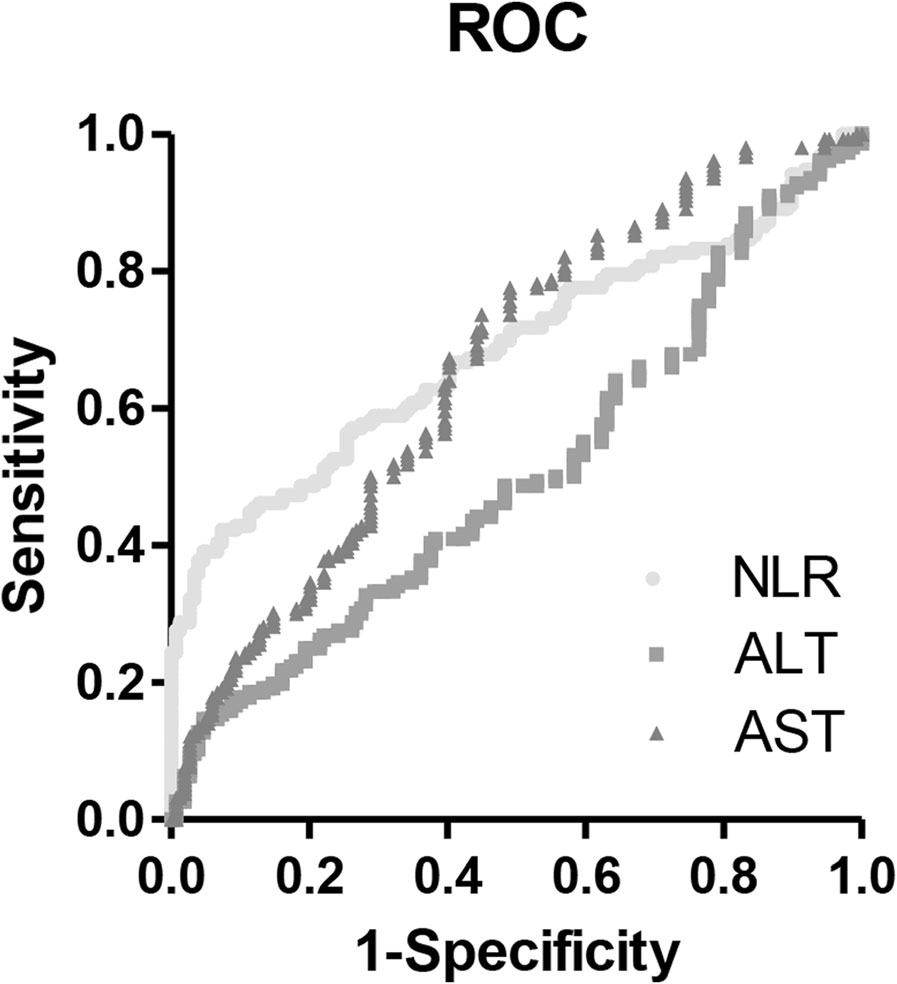
ROC curves for NLR, AST, and ALT for the diagnosis of HCC with liver disease control in patients with AFP < 20 ng/mL

In the present study, 156 (42.3%) HCC patients and 149 (85.1%) liver disease patients had an AFP level less than 20 ng/mL. We also evaluated the diagnostic accuracy of these biomarkers for HCC in these patients. As shown in Fig. 2 and Table 5, among all three biomarkers, the NLR showed the highest AUC (0.685). ALT and AST showed the same AUC and cutoff values as in the whole population, whereas the NLR had a lower AUC (0.685 vs 0.738) and a higher cutoff value (3.355 vs 2.979) with an increased specificity (0.926 vs 0.858) in patients with a low AFP level. Among biomarker combinations, NLR and ALT together showed the highest AUC (0.682) with the highest sum of sensitivity and specificity (1.423; Table 6).

**Table 5:** Diagnostic performances of three serum biomarkers for differentiating HCC from liver disease in patients with AFP < 20 ng/mL

Marker	Cutoff value	AUC	Sensitivity (Sn)	Specificity (Sp)	Sn + Sp	PPV	NPV
ALT	111	0.507 (0.449–0.564)	0.147 (0.096–0.213)	0.953 (0.906–0.981)	1.1	0.767 (0.577–0.901)	0.516 (0.456–0.577)
AST	27	0.660 (0.603–0.713)	0.981 (0.945–0.996)	0.168 (0.112–0.238)	1.149	0.552 (0.492–0.612)	0.893 (0.718–0.977)
NLR	3.355	0.685 (0.629–0.737)	0.423 (0.344–0.505)	0.926 (0.872–0.963)	1.349	0.857 (0.759–0.926)	0.605 (0.539–0.669)

HCC, hepatocellular carcinoma; ALT, alanine aminotransferase; AST, aspartate aminotransferase; AFP, alpha-fetoprotein; NLR, neutrophil-granulocyte ratio; AUC, area under the receiver operating characteristic curve

**Table 6:** Diagnostic performances of combinations of four serum biomarkers for differentiating HCC from liver disease in patients with AFP < 20 ng/mL

Markers	AUC	Sensitivity (Sn)	Specificity (Sp)	Sn + Sp	PPV	NPV
ALT+AST	0.646 (0.591–0.698)	0.737 (0.66–0.800)	0.550 (0.470–0628)	1.287	0.632 (0.560–0.699)	0.667 (0.5794–0.744)
NLR + ALT	0.682 (0.628–0.732)	0.500 (0.423–0.578)	0.873 (0.809–0.917)	1.423	0.804 (0.714–0.871)	0.645 (0.558–0.688)
NLR + AST	0.666 (0.611–0.716)	0.821 (0.753–0.873)	0.503 (0.424–0.583)	1.324	0.634 (0.565–0.697)	0.728 (0.635–0.805)
NLR + AST + ALT	0.666 (0.611–0.716)	0.821 (0.753–0.873)	0.503 (0.424–0.583)	1.324	0.634 (0.565–0.697)	0.728 (0.635–0.805)

HCC, hepatocellular carcinoma; ALT, alanine aminotransferase; AST, aspartate aminotransferase; AFP, alpha-fetoprotein; NLR, neutrophil-granulocyte ratio; AUC, area under the receiver operating characteristic curve

## Discussion

In the present study, the diagnostic values of AFP, NLR, AST, and ALT as well as their combinations were evaluated and compared. The data showed that AFP remained the best single marker, and the NLR was a comparable single marker to AFP. The combination of AFP and NLR had the best diagnostic performance (with a sum of sensitivity and specificity of 1.54) compared to all other combinations, even in patients with AFP < 20 ng/mL. Combination of three or four markers did not improve the diagnostic performance compared to the combination of AFP and NLR. In patients with AFP < 20 ng/mL, the NLR showed the best AUC as a single marker, and the combination of NLR and ALT showed the best AUC as a combination marker.

At present, early diagnosis of HCC is still a challenge. Although AFP is a well-known and widely used clinical marker for screening, diagnosing and monitoring HCC, the low sensitivity restricts its clinical application [4, 10]. Researchers are looking for new efficient diagnosis markers. microRNAs, osteopontin, glypican-3, and Cavin-2 are several biomarkers reported to be potential diagnostic indicators of HCC [4, 11, 29–31]. However, these biomarkers show limited improvement or even no improvement in HCC diagnosis compared to AFP, and they are not competitive candidates. Several studies also have investigated the combination of AFP with other biomarkers such as osteopontin, Dickkopf-1(DKK-1), protein induced by vitamin K absence (PIVKA-II) and *Lens culinaris* agglutinin-reactive fraction of AFP (AFP-L3) [10, 32]. However, the conclusions from different studies are controversial. Lim et al. found that PIVKA-II was the most accurate diagnostic marker and diagnostic accuracy was improved by combining the AFP, PIVKA-II, and AFP-L3 markers compared to each marker alone for HCC diagnosis [32]. Jang et al. found that AFP was still the most useful single biomarker and diagnostic accuracy was improved by combining AFP and DKK-1 but not other biomarkers [10]. Moreover, these added biomarkers are not common clinically measured parameters. Considering the limited diagnostic accuracy improved by combination, it is not cost-effective to introduce such new biomarkers in routine clinical detection. The diagnostic accuracy of AFP combined with some more common and available markers from routine examinations should be investigated.

The NLR is a simple biomarker of inflammation and clinically available through routine examination. It has been proposed to be of prognostic value in HCC. The NLR can predict HCC recurrence after liver transplantation or in recurrent HCC patients following thermal ablation [33, 34], and an elevated NLR indicates a poor prognosis for HCC patients [23]. All these data indicate that the NLR may reflect the disease status of the patients and may be used for screening.

In the present study, we investigated the diagnostic values of AFP, NLR, AST, and ALT alone as well as their combinations. Our data indicate that AFP is still the most effective single diagnostic marker for HCC, although the NLR is comparable to AFP. The AUC for AFP at the optimal cutoff value of 24.64 ng/mL was 0.775, which is consistent with previous reports [10, 32]. However, when applying the most common cutoff value of AFP (20 ng/mL), the AUC was only 0.664, indicating that in our study population, 20 ng/mL is not an optimal cutoff value. The AUC for the NLR in the present study was 0.738, suggesting the NLR is a promising diagnostic marker for HCC.

Further evaluation of biomarker combinations showed that the combination of AFP and NLR had the highest diagnostic accuracy, and the AUC for this combination was 0.769 with a sensitivity of 0.767 and specificity of 0.773, which showed a comparable diagnostic accuracy to the combination of AFP and DKK-1 or AFP, PICKA-II, and AFP-L3 as previously reported [10, 32]. Our data indicate that combination of AFP and NLR is a promising diagnostic marker for HCC.

When we evaluated the diagnostic value of NLR in patients with AFP < 20 ng/mL, we found that compared to AST and ALT, the NLR still showed a relative high AUC (0.685) with a sensitivity of 0.423 and specificity of 0.926, and the PPV was 0.857, indicating its possible application in this population. Further analysis of combinations of biomarkers showed that addition of other biomarkers did not improve diagnostic accuracy beyond that of NLR alone.

There are a few limitations in the present study. First, this was a retrospective study, and thus, selection bias could not be avoided. Second, only patients with liver disease caused by HBV or HCV infection were enrolled as the control group, and thus, the influencing factors may not be complex enough to reflect the whole liver disease population. Therefore, the conclusions should be further confirmed. Third, we did not collect enough data for HCC stage, and thus, the association between the screening value of AFP and HCC stage cannot be evaluated. Fourth, due to a lack of follow-up data, the impact of AFP/NLR on the development and progression of HCC over time cannot be evaluated. We will evaluate the longitudinal significance of AFP/NLR in a future study.

## Conclusions

In conclusion, a combination of AFP and NLR showed better accuracy than either marker alone for differentiating HCC from liver disease. Because the NLR is a readily measurable marker on routine examination, this study provides further insight into their clinical applications.

## Abbreviations

AFP: Alpha-fetoprotein
ALT: Alanine aminotransferase
AST: Aspartate aminotransferase
HCC: Hepatocellular carcinoma
NLR: Neutrophil-to-lymphocyte ratio
NPV: Negative predictive value
PPV: Positive predictive value
ROC: Receiver operation characteristic
